# The Estimation of Phenolic Compounds, Sugars, and Acids of the Cultivar and Clones of Red-Fleshed Apples Based on Image Features

**DOI:** 10.3390/foods14071138

**Published:** 2025-03-25

**Authors:** Ewa Ropelewska, Justyna Szwejda-Grzybowska, Mariusz Lewandowski, Monika Mieszczakowska-Frąc

**Affiliations:** 1 Fruit and Vegetable Storage and Processing Department, The National Institute of Horticultural Research, Konstytucji 3 Maja 1/3, 96-100 Skierniewice, Poland; justyna.grzybowska@inhort.pl (J.S.-G.); monika.mieszczakowska@inhort.pl (M.M.-F.); 2 Department of Horticultural Crop Breeding, The National Institute of Horticultural Research, Konstytucji 3 Maja 1/3, 96-100 Skierniewice, Poland; mariusz.lewandowski@inhort.pl

**Keywords:** red-fleshed apple, chemical properties, image parameters, correlation, regression

## Abstract

The genotypes of red-fleshed apples can vary in the intensity of red color and other fruit quality parameters. Crossing red-fleshed apple genotypes may lead to the development of new genotypes with increased health properties desired by apple processors. For fruit samples belonging to 5 genotypes, such as the cultivar ‘Trinity’ and four clones (90, 120, 156, and 158), image textures and the contents of sucrose, fructose, glucose, sorbitol, total sugars, L-ascorbic acid, malic acid, citric acid, and phenolic compounds were measured. Five groups of polyphenols, namely, flavanols, dihydrochalcones, anthocyanins, flavonols, and phenolic acids, were determined. The correlations between the chemical and image properties of apple samples were determined. The regression equations to estimate the chemical compounds content in red-fleshed apple samples based on image features were set. Generally, the results revealed that red-fleshed apple clones and a cultivar can statistically significantly differ in the content of phenolic compounds, sugars, and acids. Strong relationships between all examined chemical parameters with selected image texture features were observed. The highest correlation coefficients were found between citric acid with texture ZS5SH3Correlat (R = −0.999), total flavanols with RS5SH5Correlat (R = 0.999), quercetin-xyloside (group of flavonols) with XS5SH5Entropy (R = 0.999), and total sugars with GS5SH1SumVarnc (R = −0.998). The developed regression equations allowed for correct estimations of acid, sugar, and phenolic compound contents based on image textures with the coefficients of determination (R^2^) reaching 0.998 for citric acid, total flavanols, and quercetin-xyloside, and 0.996 for total sugars.

## 1. Introduction

Apple is one of the most economically important tree species grown around the world, and it is well adapted to cultivation in the natural conditions of Poland. World apple production in 2024 amounted to about 84 million tons [1]. According to the U.S. Department of Agriculture estimates, apple production in Poland amounted to about 3.4 million tons in the same period. Poland is, therefore, the largest producer of this species of fruit in the European Union and the fourth in the world after China (48 million tons), the United States (4.9 million tons), and Turkey (4.2 million tons). Apple fruits have many benefits for human health and are a favorite fruit among consumers [2,3,4].

In recent years, apples with red flesh have become increasingly popular, which, unlike white-fleshed apples, contain anthocyanins throughout the fruit [5,6,7]. Studies have shown that anthocyanins and other polyphenolic compounds have various biological properties that can counteract the aging of brain nerves, strengthen immunity, lower blood sugar and lipid levels, and also have antioxidant and anticancer effects [6,8,9]. Therefore, the cultivars of these apples, not only because of their attractive appearance, have become the subject of interest of both many researchers and processors [5,6,7,10]. Many consumers are not yet aware of the existence of such apple cultivars that can be used to create new products with high health-promoting value. Research is being conducted in many countries on their cultivation, but commercial orchards are uncommon.

Red-fleshed apples, unlike white-fleshed cultivars, contain anthocyanins in the flesh, which have health-promoting properties [11]. They can be an interesting raw material for cloudy apple juice production. Juices from apple cultivars with slightly red flesh are refreshing and can be an attractive product for consumers. Currently, there are several or even several dozen cultivars with red flesh available on the market (‘Maypole’, ‘Pomfital’, ‘Trinity’, ‘Redlove^®^’, ‘Bloody Ploughman’, ‘Grenadine’, ‘Pink Pearmain’, ‘Rubens Lilliputapfel’, ‘Vitalstar’, and ‘Thornberry’). Characteristic features of these cultivars are high acidity and high vitamin C content. These cultivars are resistant to apple scab, which facilitates their production. Apple fruits with red flesh can be grown both for desserts and for processing, for example, for juice production [11].

The genotypes of red-fleshed apples from the *Malus* are diverse in terms of the intensity of red color and its distribution and may probably come from the *M. sieversii* species, which naturally occurs in central Asia [11]. In the studies of Lin-Wang et al. [12], it was found that there are the following two types of red-fleshed apples: one caused by the MdMYB10 gene on chromosome (ch.) 9 and the second one caused by the MdMYB110a gene on ch. 17. In turn, MdMYB10 is an analyte of MdMYBA/MdMYB1, which is responsible for the color of the fruit skin. Wang [13] stated that the genetic background of red-fleshed apples is complicated and a mutation of a transcription factor or structural gene may affect the red-fleshed phenotype. Studies of the species *M. sieversii* indicated the existence of large differences, both in fruit quality (color, palatability, and morphology) and in relation to the phenotypic features of trees (height and shape). It was also found that this species has developed resistance to drought, cold, and pests. Therefore, it has aroused increasing interest among breeders [13,14]. Crossing current genotypes of red-fleshed apples can become not only an attractive point for developing new genotypes with increased health properties but also for processors looking for new, original, and valuable raw materials. Therefore, in-depth research on new clones is essential.

The approach to assessing the quality of red-fleshed apples applied in this study combines the analysis of chemical properties with image analysis of newly developed clones and one known cultivar. This study aimed at determining the relationships between the contents of sucrose, fructose, glucose, sorbitol, total sugars, citric acid, malic acid, L-ascorbic acid, and phenolic compounds (flavanols, dihydrochalcones, phenolic acids, flavonols, and anthocyanins) and image textures of red-fleshed apples belonging to five genotypes, such as the cultivar ‘Trinity’ and clones 90, 120, 156, and 158. Innovative aspects that were not raised in previous studies concern finding image parameters among over 2000 textures that would be correlated with selected chemical properties of examined samples. The pioneering nature of the research concerns the development of regression equations to estimate the content of chemical properties in red-fleshed apples based on image textures. The innovation is the use of newly produced red-fleshed apple clones, which are heading towards the development of dessert cultivars. The development of an approach based on image textures determined in an objective and efficient manner for estimating the chemical quality of new apple clones is a great novelty in breeding programs. The quantitative analysis of textures extracted from digital color images can provide insights into apple quality. Images of the flesh of different clones can exhibit different subpatterns of the dispersion and distribution of pixel brightness, which represent the color, smoothness, roughness, size, directivity, and granulation of textures. These patterns indicate the structure of the apple flesh, which also depends on chemical properties. Therefore, it was assumed that the chemical properties of red-fleshed apples can be predicted from the texture image of the flesh.

## 2. Materials and Methods

### 2.1. Red-Fleshed Apple Samples

One of the aims of the apple breeding program conducted at the National Institute of Horticultural Research is to develop new genotypes that are either resistant or show low susceptibility to apple scab (*Venturia inaequalis*), apple powdery mildew (*Podosphaera leucotricha*), and fire blight (*Erwinia amylovora*). New cultivars should produce high yields of good fruit quality (with red flesh and high anthocyanin content) and should be well adapted to climatic conditions of Poland. Cultivation of such cultivars is enabling the production of apples without or with very low levels of chemical residues harmful to human health at markedly reduced production costs. Apples were sampled from the apple collection at the Pomological Orchard of the National Institute of Horticultural Research (InHort), Skierniewice, central Poland. Each genotype/clone in our collection is represented by 3 trees growing on the M.9 rootstock. The raw material of apples belonged to the following five genotypes: ‘Trinity’, as a standard cultivar, clone numbered 90 with ‘Trinity’ used as the paternal (pollen) in the crossbreeding, clone numbered 120 with ‘Trinity’ also used as the paternal (pollen) in the crossbreeding, clone numbered 156 with the origin of ‘Ligol Red’ × ‘Trinity’, and clone numbered 158 with the origin of ‘Roxana’ × ‘Trinity’. All clones (90, 120, 156, and 158) were obtained from a crossbreeding program performed in 2009. The two clones, 90 and 120, came from the same hybrid family, in which the parental form (mother) used clone J-79 (genotype resistant to apple scab and with very low susceptibility to powdery mildew and fire blight with white flesh). The general characteristics of these genotypes in terms of growth strength, fruit appearance, fruit storage, and tree susceptibility to diseases were presented in the publication by Ropelewska and Lewandowski [15]. For each clone (90, 120, 156, and 158) and a cultivar ‘Trinity’, 25 apple samples were used to analyze in this study.

### 2.2. Image Parameters

**Figure 1:**
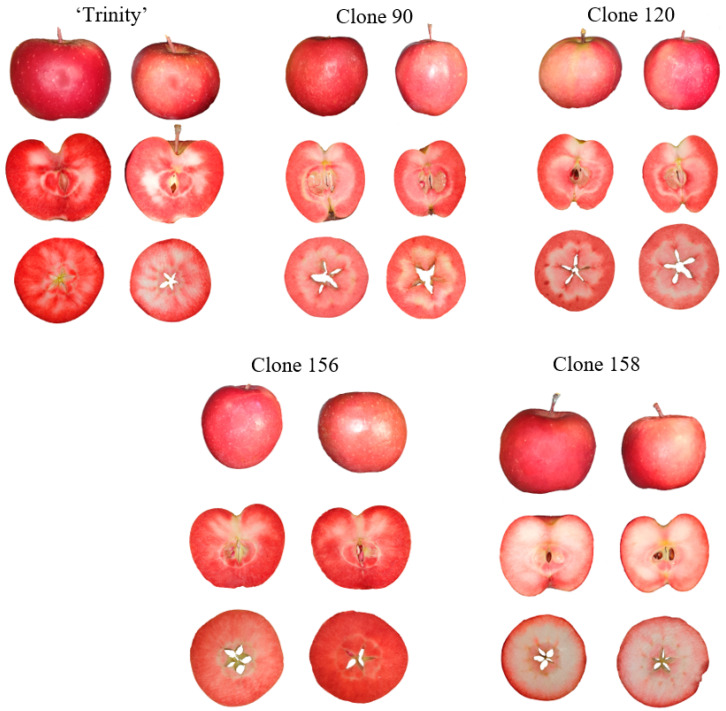
Images of whole samples, longitudinal sections, and cross-sections of red-fleshed apples of the cultivar ‘Trinity’ and clones 90, 120, 156, and 158.

The image acquisition of apple samples by a flatbed scanner was performed using an Epson Perfection (Epson, Suwa, Nagano, Japan). Before imaging, red-fleshed apples belonging to the cultivar ‘Trinity’ and four clones were cut into 4 mm thick slices. For each of the 25 apples of each clone and a cultivar, 4 slices were obtained. Therefore, in total, images of one hundred apple slices for each clone of 90, 120, 156, and 158 and a cultivar ‘Trinity’ were obtained. Slices were imaged on a black background. The apple images were saved as a .tiff extension. The resolution of the images was 1200 dpi. The exemplary images of apple samples are presented in Figure 1. The image processing was performed using the MaZda 4.7 software (Institute of Electronics, Łódź University of Technology, Łódź, Poland) [16,17,18]. The image segmentation was performed using brightness thresholding. Lighter slices with higher pixel values were extracted from the darker background. Each slice was a region of interest (ROI). For each slice image in color channels *X*, *Y*, *Z*, *L*, *a*, *b*, *R*, *G*, *B*, *S*, *V*, and *U*, 2172 image textures were computed. The extracted image textures were considered as a function of the spatial variation in the pixel brightness intensity. After imaging, the same apple samples were subjected to chemical analysis.

### 2.3. Chemical Properties

For the analysis of the concentration of sugar compounds, organic acids, and polyphenol compounds, apple slices were ground in dry ice, after which they were disintegrated and stored at −20 °C for the analysis.

#### 2.3.1. Sugar Content

HPLC analysis of the individual sugars in apples was performed by an Agilent 1200 HPLC system (Agilent Technologies, Morges, Switzerland) equipped with a differential refractometric detector. The separation of sugars was carried out using a column (Aminex HPX-87C (300 mm × 7.5 mm) (Bio-Rad Laboratories, Hercules, CA, USA) with a precolumn and the temperature maintained at 80 °C at a flow rate of 0.6 mL min^−1^. As a mobile phase, edetate calcium disodium (Ca-EDTA) (Sigma-Aldrich Chemie GmbH, Steinheim, Germany) was used in isocratic flow. Samples for sugar determinations were dissolved in deionized water, homogenized, and filtrated through a SepPak PLUS C18 filter into vials. Sucrose, glucose, fructose, and sorbitol standards (Sigma-Aldrich Chemie GmbH, Steinheim, Germany) were prepared as external standards for identification and quantification. The sugars were quantified by a calibration curve for individual sugars. Total sugars were calculated as the sum of individual sugars. Data are the mean of three replicates and expressed as g of total sugar content per kg fm.

#### 2.3.2. Analysis of Acids

An Agilent 1200 HPLC system equipped with a Diode Array Detector (DAD) was used to identify and quantify organic acids using a Supelcosil LC-18 column (250 mm × 4.6 mm; 5 µm) (Sigma-Aldrich Chemie GmbH, Darmstadt, Germany) with a precolumn. A 1% phosphate-buffered solution, KH_2_PO_4_, pH 2.5, (potassium phosphate, monobasic, JT Baker Chemicals, Phillipsburg, NJ, USA) was used as a mobile phase in an isocratic system. The column temperature was kept at a temperature of 30 °C and a flow rate of 0.8 mL min^−1^. The detection of L-ascorbic acid was performed by absorbance at 244 nm for L-ascorbic acid and 210 nm for malic and citric acids (Sigma-Aldrich Chemie GmbH, Steinheim, Germany). The samples for analysis were dissolved in 6% HPO_3_ (meta-phosphoric acid, Sigma-Aldrich Chemie GmbH, Steinheim, Germany), homogenized, and filtered. The acids were quantified by a calibration curve for malic, citric, and L-ascorbic acids. The results of three replicates were expressed in milligrams per 100 g fm. 

#### 2.3.3. Phenolic Compounds Analysis

Determination of the concentration of individual phenolic compounds was performed according to the described method by Tsao and Yang [19] Quantitative analysis of major phenolic compounds was performed on the Agilent 1200 series HPLC system equipped with a DAD detector (Sigma-Aldrich Chemie GmbH, Darmstadt, Germany). The separation of compounds was carried out using a Synergi 4 μm Fusion-RP 80A column (250 mm × 4.6 mm) with a guard column (Phenomenex, Torrance, CA, USA). Two solvents were used for the separation in a gradient system. The mobile phase was a solution of 2 mM sodium acetate (JT Baker Chemicals, Phillipsburg, NJ, USA) containing 10.2% acetic acid (solvent A) and acetonitrile (solvent B) (JT Baker Chemicals, Phillipsburg, NJ, USA). Conditions included 25 °C and a flow rate of 0.5 mL min^−1^. The separation was performed using the following steps: 0–20 min, 3% B linear; 20–40 min, 17% B linear; 40–65 min, 40% B linear; 65–68 min, 90% B linear; 68–72 min, 90% B isocratic; and 72–73 min, 0% B linear, followed by washing and reconditioning of the column. The content of individual compounds monitoring was performed at wavelengths of 280 nm (epicatechin, procyanidins oligomers, phloridzin, and florentine xyloside), 320 nm (chlorogenic acid and derivatives chlorogenic acid), 360 nm (quercetin-3-galactoside, quercetin-3-glucoside, quercetin-3-xyloside, quercetin-3-arabinoside, and quercetin-3-rhamnoside), and 520 nm (cyanidin-3-galactoside). Samples for polyphenol determinations were homogenized in 70% methanol (JT Baker Chemicals, Phillipsburg, NJ, USA). The supernatant was then filtered through a 0.45-μm syringe filter prior to subsequent analysis. Peaks were identified by comparing retention times and UV spectra with authentic standards (Extrasynthese, Genay, France)). The amount of individual polyphenols was calculated from the calibration curves. The calibration curve was plotted for each standard. The content of individual phenolic compounds of three replicates was expressed in milligrams per 100 g fm.

### 2.4. Statistical Analysis

The statistical analysis, including mean comparison, correlation analysis, and regression analysis, was carried out using STATISTICA 13.1 (Dell Inc., Tulsa, OK, USA). First, the mean values of the content of sugars, acids, phenolic compounds, and selected image textures of the cultivar ‘Trinity’ and clones 90, 120, 156, and 158 were compared to determine the statistically significant differences between samples. Analysis was performed at a significance level of *p* < 0.05 using Tukey’s test after checking the homogeneity of variance and normality of the distribution of variables. Then, the linear relationships of image features and chemical parameters of apple samples based on Pearson’s correlation coefficients (R) were determined. The significance of the observed correlations was determined at a significance level of *p* < 0.05. The confidence intervals were 95%. As a result, for each chemical parameter, one strongest positive correlation and one strongest negative correlation were selected based on the highest values of R. For these relationships, the regression equations were determined to estimate the content of sugars, acids, and phenolic compounds based on image textures. Additionally, the scatter plots were created to present the relationships between selected image features and chemical characteristics of red-fleshed apples.

It was assumed that the chemical properties of red-fleshed apples depended on the cultivar and clones, and then these properties influenced the flesh structure reflected in the image textures. Therefore, the Principal Component Analysis (PCA) was performed using STATISTICA 13.1 to indicate the relationships between the cultivar and clones and chemical parameters of samples. The multivariate PCA was used to determine relationships between the cultivar ‘Trinity’ and clones 90, 120, 156, and 158 and sucrose, fructose, glucose, sorbitol, total sugars, L-ascorbic acid, malic acid, and citric acid, as well as the cultivar ‘Trinity’ and clones 90, 120, 156, and 158 and flavanols, dihydrochalcones, anthocyanins, flavonols, and phenolic acids. The PCA graphs were created.

## 3. Results and Discussion

**Figure 2:**
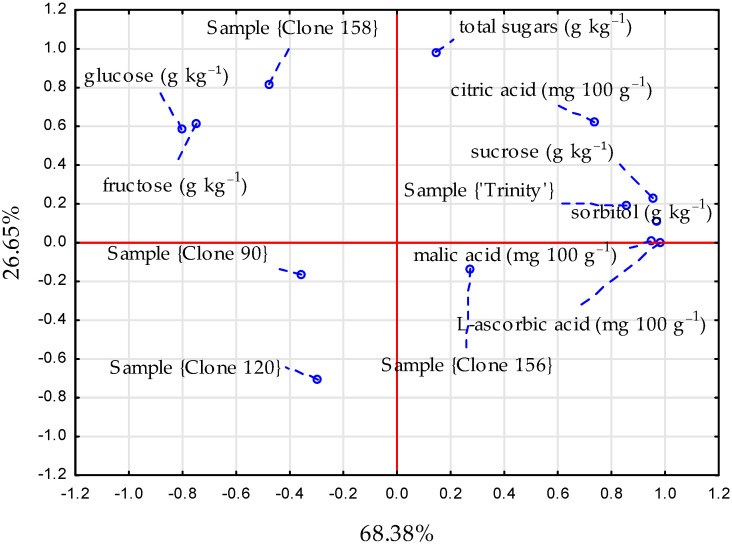
The PCA graph illustrating the relationships between the type of red-fleshed apples (cultivar ‘Trinity’ and clones 90, 120, 156, and 158) and sugar and acid contents, blue-dashed line—line connecting the point with the name.

The red-fleshed apple flesh is rich in components, such as sugars and acids [20]. The content of sugar components and organic acids is mainly determined by the apple genotype [21,22,23] and depends on environmental and agrotechnical factors [24]. The content of these components in the red-fleshed apple samples is shown in Table 1, and the relationships between the type of apples and the sugar and acid contents are presented in Figure 2.

The quantitative composition of sugars in the tested apple samples is presented in Table 1. The tested red-fleshed apples contained disaccharide—sucrose, monosaccharides—glucose and fructose, and sugar alcohol—sorbitol. The total sum of sugar components in the ‘Trinity’ cultivar was (102.0 g kg^−1^ f.m.), while in the apple clones, it was 85.9–108.6 g kg^−1^ f.m. The dominant sugar in the ‘Trinity’ apple cultivar was sucrose, which constituted 47.2% of all sugar components. In the studies by Ropelewska et al. [25], the highest share of sucrose was also found in red-fleshed apples of the following cultivars: ‘Trinity’, ‘Roxana’, and ‘Alex Red’. In turn, Mieszczakowska-Frąc [10] determined a similar level of this sugar in three consecutive years in the ‘Trinity’ cultivar. In red-fleshed apple clones, the dominant sugar was fructose, the share of which was 51.2–66.1%. Glucose accounted for 5–15.2% of all sugars. The highest sorbitol content was recorded in the control cultivar ‘Trinity’ (6.68 g kg^−1^ f.m.). A high sorbitol content in the ‘Trinity’ cultivar was observed in the studies by Ropelewska et al. [25] at two harvest dates.

Red-fleshed apples contain various organic acids, but the dominant one is malic acid, which was also indicated by other researchers [10,20,25,26]. In our studies, the content of malic acid was high, and for the ‘Trinity’ cultivar, it amounted to 1578 mg·100 g^−1^ f.m., while in the clones, it ranged from 843 to 1449 mg 100 g^−1^ f.m. (Table 1). The red-fleshed apple clones contained approximately 35–50% less malic acid compared to the control cultivar ‘Trinity’. The exception was clone 156, in which the content of this acid was at a similar level (1449 mg 100 g^−1^ f.m.). The literature data indicated that the ‘Trinity’ cultivar may contain higher contents of malic acid (1724–1970 mg 100 g^−1^ f.m.) depending on the season [10]. Our study showed that the red-fleshed apple clones varied in the content of L-ascorbic acid, the content of which in the tested clones was in the range of 1.88–5.84 mg 100 g^−1^ f.m. The control cultivar ‘Trinity’ contained approximately 35–80% more of this component (9.20) mg 100 g^−1^ f.m. In a 3-year study, Mieszczakowska-Frąc [10] determined the level of L-ascorbic acid at 10.8–15.9 mg 100 g^−1^ f.m. in the ‘Trinity’ cultivar. The content of L-ascorbic acid in the skin and flesh of apples depends strictly on the cultivar and does not depend on the harvest date [27]. Citric acid was present at a concentration similar to that given in other studies [10] and was clearly higher in the ‘Trinity’ control cultivar (17.67 mg 100 g^−1^ f.m.) compared to red-fleshed clones (10.27–15.09 mg 100 g^−1^ f.m.). In addition, the levels of acids in apples depend on the balance between the biosynthesis of organic acid biosynthesis, degradation, and vacuolar storage [28].

**Figure 3:**
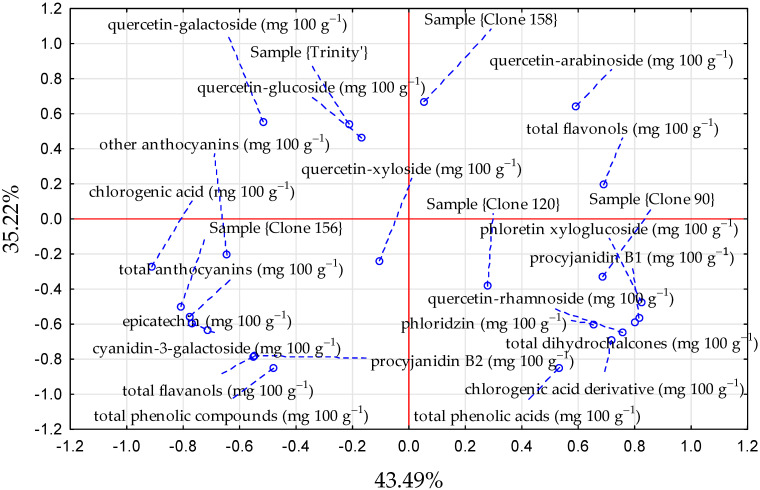
The PCA graph illustrating the relationships between the type of red-fleshed apples (cultivar ‘Trinity’ and clones 90, 120, 156, and 158) and polyphenolic compound contents, blue-dashed line—line connecting the point with the name.

Red-fleshed apples, unlike white-fleshed cultivars, are characterized by a high content of anthocyanins and other polyphenolic compounds that have health-promoting properties [13,29,30]. The content of individual polyphenolic compounds in red-fleshed apple samples is presented in Table 2, and the relationships between the type of apples and the polyphenolic compound contents are presented in Figure 3. The average content of polyphenolic compounds in the examined red-fleshed apple clones was in the range of 32.91–99.70 mg 100 g^−1^ f.m., and in the control cultivar ‘Trinity’, it was 56.14 mg 100 g^−1^ f.m. In our study, the following five classes of phenolic compounds were found in red-fleshed apple samples: flavanols (procyanidin oligomers, (-)-epicatechin), dihydrochalcones (phloretin xyloglucoside and phloridzin), phenolic acids (mainly chlorogenic acid), flavonols (quercetin glycosides), and anthocyanins (mainly cyanidin-3-galactoside), which is confirmed by the available literature [25,31,32,33]. In the study by Mieszczakowska-Frąc [10], a similar profile of polyphenolic compounds was observed in the ‘Trinity’ cultivar in three experimental seasons. In our study, the dominant group of compounds in the ‘Trinity’ cultivar and red-fleshed apple clones were anthocyanins (mainly cyanidin-3-galactoside), the content of which is characteristic for red-fleshed apples. The average content of anthocyanins in the tested apple clones ranged from 13.52 to 64.47 mg 100 g^−1^ f.m., and in the control cultivar ‘Trinity’ 37.52 mg 100 g^−1^ f.m. Significantly, the highest content was characteristic for the clone 156, while the lowest was characteristic for the clone 158. The shares of anthocyanins in the total phenolic compounds of the tested apples were as follows: ‘Trinity’ cultivar—66.8%, clone 90—32.9%, clone 120—51.6%, clone 156—64.7%, and clone 158–41%. In addition to cyanidin-3-galactoside, other anthocyanins may also be present in red-fleshed apples, such as cyanidin-3-glucoside, cyanidin-3-arabinose, cyanidin-7-arabinose, xyloside-cyanidin, and peonidin-3-galactoside, which was confirmed in other studies [32,34].

Flavanols are the flavonoid pathway products, which also lead to the formation of anthocyanins and flavonols [35]. In the group of flavanols, the following two procyanidin oligomers were identified in apple samples: B1 and B2, and one monomer-(-)-epicatechin (Table 2). Mieszczakowska-Frąc [10], in a 3-year study in the ‘Trinity’ cultivar, also observed only the presence of (-)-epicatechin and procyanidin oligomers. In the present study, procyanidin B1 was present only in the following two apple clones: 90 and 120. Significantly, the highest content of flavanols was noted in clone 156 (14.6 mg 100 g^−1^ f.m.), in an amount 2–4 times higher than in the others. In the studies by Sato et al. [36], the presence of other flavanols, e.g., (+)-catechin, was also confirmed in four apple cultivars with red flesh.

The main phenolic acid present in the tested red-fleshed apple samples was chlorogenic acid, the content of which varied from 4.38 mg 100 g^−1^ to 16.8 mg 100 g^−1^ f.m (Table 2). The highest level of chlorogenic acid was recorded in clone 90 and clone 120, while the lowest was the ‘Trinity’ cultivar. In clone 156 and the ‘Trinity’ cultivar, small amounts of a chlorogenic acid derivative were additionally identified (1.14–3.30 mg 100 g^−1^ f.m.). Studies by other authors also confirm the occurrence of one hydroxycinnamic acid (chlorogenic acid) in red-fleshed apple cultivars [10,32,37].

Quercetin glycosides were the only flavonols identified in the apple samples in this study (Table 2). Their content in the apple clones was at a similar level and ranged from 3.58 to 5.75 mg 100 g^−1^ f.m., and in the ‘Trinity’ cultivar, it was 5.13 mg 100 g^−1^ f.m.

Dihydrchalcones were also determined in the red-fleshed apple samples tested (phloretin xyloglucoside and phloridzin) (Table 2). Phloridzin was the dominant dihydrochalcone in all apple samples, and its content for the clones ranged from 4.67 to 8.14 mg 100 g^−1^ f.m. and in the ‘Trinity’ cultivar was 2.59 mg 100 g^−1^ f.m. The highest contents of dihydrochalcones of 13.0 mg 100 g^−1^ f.m. and 10.27 mg 100 g^−1^ f.m. were determined for clones 90 and 120, respectively, and the lowest of 4.63 mg 100 g^−1^ f.m for the ‘Trinity’ cultivar. Wang et al. [32] and Mieszczakowska-Frąc [10], examining various cultivars of red-fleshed apples, also noted its higher share in red-fleshed apples. Tsao et al. [19] found that although dihydrochalcones are generally present in apples in small amounts, apple cultivars can be distinguished based on their content [19].

Studies in the available literature confirmed the large diversity of polyphenolic compounds depending on the red-flesh apple cultivar and their seasonal variability. Environmental factors such as light intensity, climatic conditions, pH, water deficiency, and storage conditions may also be associated with the synthesis of anthocyanins and other classes of polyphenols in red-flesh apples [38,39,40,41,42]. In addition, Juhart et al. [41] found that transcription factors are responsible for the anthocyanin biosynthesis. During the ripening of red-fleshed apples, there is a strong association between overexpression of the MdMYB10 gene and the expression of anthocyanin levels, which induce the red color of the apple flesh.

The sugars, acids, and phenolic compound contents presented in Table 1 and Table 2, respectively, were used to correlate with selected image textures. Therefore, the mean values of these textures of red-fleshed apples of the cultivar and clones are presented in Table 3. It was found that also image texture parameters differ depending on the type of apples.

The relationships of chemical properties with the image textures were determined. Strong correlations of image features with sugars and acids of fleshed apples of the cultivar ‘Trinity’ and clones 90, 120, 156, and 158 were observed (Table 4). The content of sucrose, glucose, fructose, sorbitol, and total sugars was positively and negatively correlated with selected image textures. The highest positive correlation coefficient of 0.996 was found between sucrose (g kg^−1^) and image texture LS5SH3DifVarnc, and the highest negative correlation coefficient of −0.998 was between total sugars (g kg^−1^) and texture GS5SH1SumVarnc. In the case of acids, the strongest correlations were determined for citric acid (mg 100 g^−1^) with LS5SV3Contrast (R = 0.995) and ZS5SH3Correlat (R = −0.999).

Very strong relationships were also found between image features and the content of phenolic compounds (mg 100 g^−1^) of red-fleshed apples (Table 5). The values of correlation coefficients reached 0.999 for the content of total flavanols with image texture RS5SH5Correlat and quercetin-xyloside (a group of flavonols) with XS5SH5Entropy. The highest negative correlation with R equal to −0.998 was determined between the content of chlorogenic acid derivative (group of phenolic acids) and image texture aS5SH5SumOfSqs. In the case of dihydrochalcones, the highest R of 0.989 was found between phloretin xyloglucoside and bS5SN1Correlat. In the group of anthocyanins, the strongest correlations were observed for cyanidin-3-galactoside with bATeta1 (R = 0.988) and with UHPerc99 (R = −0.988).

In the case of the strongest relationships with the highest R for individual chemical properties with selected image textures, the regression equations and coefficients of determination (R^2^) were determined. In the case of sugars and acids (Table 6), the developed equations can allow for correct estimations of these properties based on image textures. The values of R^2^ were very high, and in the case of sugars, it reached 0.996 for total sugars, 0.994 for sorbitol, and 0.992 for sucrose. Among acids, the highest R^2^ of 0.998 was found for citric acid.

**Figure 4:**
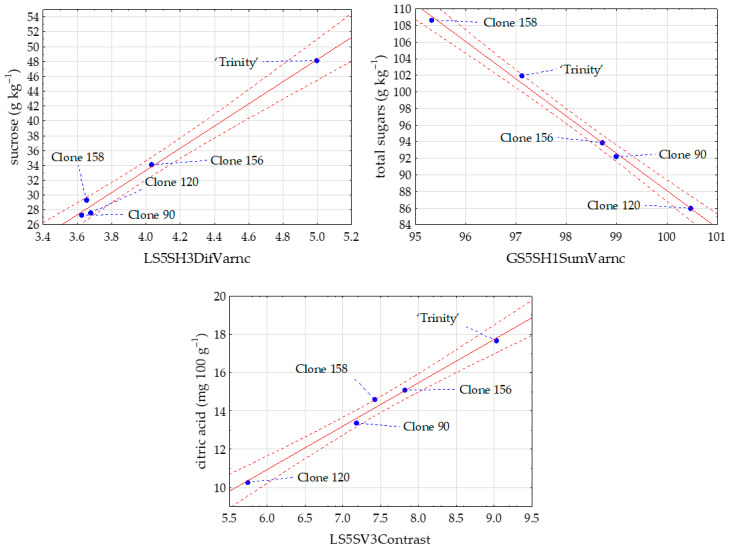
Scatter plots for image texture parameters with sugars of red-fleshed apples belonging to the cultivar ‘Trinity’ and clones 90, 120, 156, and 158, solid red line—regression line, red-dashed line—confidence interval (95%).

To visualize the relationships between image textures and sugars of examined red-fleshed apples, scatter plots for the strongest correlations are presented (Figure 4). Graphs show the strongest positive correlation between sucrose and the image texture parameter LS5SH3DifVarnc, as well as the highest negative correlations for total sugars with GS5SH1SumVarnc and citric acid with ZS5SH3Correlat. It is visible that the obtained data for a cultivar ‘Trinity’ and clones 90, 120, 156, and 158 are on the lines or very close to the lines, which indicates very strong linear correlations between examined parameters.

The regression equations were also determined for the strongest relationships of phenolic compounds with image textures of red-fleshed apples of the cultivar ‘Trinity’ and clones 90, 120, 156, and 158 (Table 7). The highest values of R^2^ were observed for total flavanols (0.998), quercetin-xyloside (0.998), procyanidin B2 (0.996), chlorogenic acid derivative (0.995), and total flavonols (0.994).

**Figure 5:**
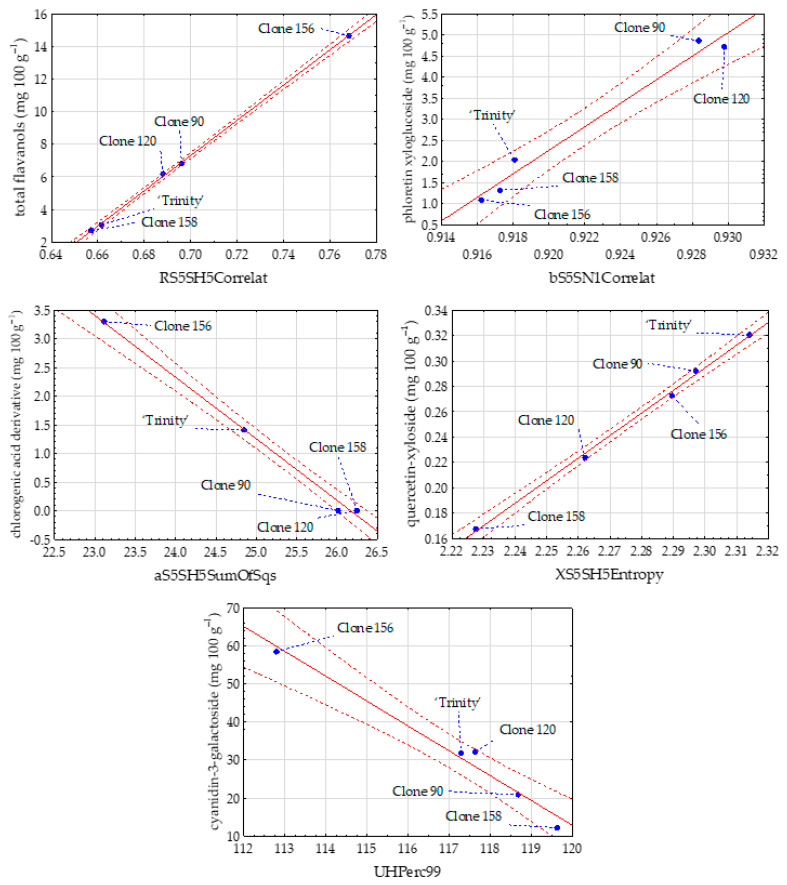
Scatter plots for image texture parameters and phenolic compounds of red-fleshed apples of the cultivar ‘Trinity’ and clones 90, 120, 156, and 158 solid red line—regression line, red-dashed line—confidence interval (95%).

In the case of each group of phenolic compounds, such as flavanols, dihydrochalcones, phenolic acids, flavonols, and anthocyanins, one scatter plot for the strongest relationship between phenolic compounds and image textures of red-fleshed apple samples was created (Figure 5). It was for total flavanols with RS5SH5Correlat, phloretin xyloglucoside with bS5SN1Correlat, chlorogenic acid derivative with aS5SH5SumOfSqs, quercetin-xyloside with XS5SH5Entropy, and cyanidin-3-galactoside with UHPerc99.

The research performed on red-fleshed apples may be of great commercial importance in the future. There are few studies on red-fleshed apples, which may be of great commercial importance in the future. There is a growing interest in these varieties worldwide due to their health-promoting properties. In recent years, scientists have increasingly emphasized the health-promoting effects of phenolic compounds and encouraged consumers to enrich their daily diet with foods containing these valuable antioxidants. The high content of anthocyanins in red-fleshed apples is very beneficial due to their antioxidant, anticancer, and antiaging properties [43,44].

The obtained results confirmed that image features depend on the apple genotype and are correlated with chemical properties, which are also genotype-dependent. The determined image texture parameters, as functions of the spatial variation in the pixel brightness intensity, provide information about the examined object structure [16]. In the previous studies, it was found that image textures are useful for discriminating the apple cultivars [45,46]. Our studies showed that differences in the chemical composition of red-fleshed apple clones and a cultivar were also reflected in differences in the structure of the samples. Therefore, the estimation of chemical compounds using models based on image textures can be possible. Also, in the available literature, the possibility of the prediction of physical and chemical properties of fruit samples was reported. For example, Shahedi [47] predicted the total anthocyanin content, weight loss, and firmness of sweet cherries using image parameters and artificial neural network (ANN) models. In the case of fresh citrus fruit, ANN and multiple linear regression (MLR) models were used to estimate the total soluble solids, vitamin C, acidity, total sugars, and reducing sugars using the fruit length, diameter, weight, and yield [48]. Whereas Mohammed et al. [49] applied ANN models to predict date fruit quality attributes such as total soluble solids, pH, moisture content, and water activity using electrical properties.

In our study, regression models turned out to be useful for estimating the chemical quality of red-fleshed apples based on image characteristics. Very strong relationships between chemical properties and image textures were revealed. The differences in chemical characteristics can also be reflected in the color and structure of apples, which affect the image textures of the flesh. The coefficients reaching 0.999 confirmed these relationships, indicating close to perfect linear correlations. However, when two variables are strongly correlated, further research is needed to confirm the obtained trends. Therefore, the future experiments can be extended to the use of samples belonging to other cultivars and collected in other growing seasons to check the developed regression equations. Furthermore, in addition to applied regression models, shallow and deep neural network models can also be used.

## 4. Conclusions

Crossing different genotypes of red-fleshed apples with another genotype can lead to the creation of a cultivar with a higher content of bioactive compounds, which was shown in our studies. Examples are clone 156, where almost a double increase in polyphenol content was observed compared to the control cultivar ‘Trinity’. In addition, red-fleshed apples are characterized by a high content of organic acids, which makes them sour, and this in turn may affect their acceptability to consumers. In our studies, it was observed that the combination of different genotypes of red-fleshed apples led to a decrease in the acid content and an increase in the fructose content, which is much sweeter than sucrose. Perhaps, thanks to this, it will be possible to obtain apple cultivars with potential pro-health benefits and desirable taste values. Moreover, it was shown that the chemical properties of red-fleshed apples can be estimated based on image parameters. The obtained results may be of great practical importance for breeders and processors of red-fleshed apples.

In addition, breeding new apple genotypes is very important for the development of fruit growing for several reasons. Apple varieties may differ in susceptibility to diseases, cultivation technology, yield efficiency, and fruit size. In order to maintain the volume and quality of production, old trees must be replaced with new ones, perhaps attractive red clones of a given variety or another that is less susceptible to diseases. Fruit growers also decide to change varieties for economic reasons when the production of apples of a particular variety becomes less and less profitable. Another reason for breeding new varieties is the need to adapt to the tastes and requirements of consumers. Many of them are looking for new taste experiences. The diversity of cultivars may encourage greater consumption of apples.

## Figures and Tables

**Table 1:** The content of sugars and acids (mean values ± standard deviations) of red-fleshed apples belonging to the cultivar ‘Trinity’ and clones 90, 120, 156, and 158.

Properties		Apple Sample				
		‘Trinity’	Clone 90	Clone 120	Clone 156	Clone 158
Sugars	Sucrose (g kg^−1^)	48.15 ± 0.12 a	27.34 ± 0.34 d	27.59 ± 0.29 d	34.08 ± 0.16 b	29.30 ± 0.13 c
	Glucose (g kg^−1^)	5.11 ± 0.15 a	11.45 ± 0.02 d	8.12 ± 0.02 c	6.35 ± 0.02 b	16.53 ± 0.31 e
	Fructose (g kg^−1^)	42.03 ± 0.17 a	49.25 ± 0.34 d	46.86 ± 0.25 b	48.1 ± 0.28 c	59.27 ± 0.23 e
	Sorbitol (g kg^−1^)	6.68 ± 0.31 a	4.17 ± 0.02 c	3.38 ± 0.13 d	5.38 ± 0.04 b	3.51 ± 0.01 d
	Total sugars (g kg^−1^)	102.0 ± 0.74 b	92.21 ± 0.72 c	85.94 ± 0.43 d	93.91 ± 0.18 c	108.6 ± 0.22 a
Acids	L-ascorbic acid (mg 100 g^−1^)	9.20 ± 0.06 a	1.88 ± 0.16 d	3.76 ± 0.01 c	5.84 ± 0.26 b	2.42 ± 0.29 d
	Malic acid (mg 100 g^−1^)	1578 ± 0.18 a	843 ± 0.62 c	1048 ± 0.24 b	1449 ± 0.01 a	954 ± 0.23 bc
	Citric acid (mg 100 g^−1^)	17.67 ± 0.29 a	13.36 ± 0.84 b	10.27 ± 0.50 c	15.09 ± 0.50 b	14.58 ± 0.46 b

a,b,c,d,e—The same letters in the rows mean no statistical differences between apple samples.

**Table 2:** The content of phenolic compounds (mean values ± standard deviations) of red-fleshed apples of the cultivar ‘Trinity’ and clones 90, 120, 156, and 158.

Phenolic Compounds		Apple Sample				
		‘Trinity’	Clone 90	Clone 120	Clone 156	Clone 158
Flavanols	Procyanidin B1 (mg 100 g^−1^)	0.00 ± 0.00 a	1.43 ± 0.21 b	1.12 ± 0.06 b	0.00 ± 0.00 a	0.00 ± 0.00 a
	Procyanidin B2 (mg 100 g^−1^)	2.18 ± 0.28 a	3.45 ± 0.18 b	3.01 ± 0.13 b	6.14 ± 0.18 c	1.75 ± 0.14 a
	Epicatechin (mg 100 g^−1^)	0.89 ± 0.01 a	1.92 ± 0.15 c	2.05 ± 0.15 c	8.51 ± 0.21 d	0.95 ± 0.15 b
	Total flavanols (mg 100 g^−1^)	3.06 ± 0.29 a	6.80 ± 0.54 b	6.18 ± 0.23 b	14.65 ± 0.40 d	2.70 ± 0.29 a
Dihydrochalcones	Phloretin xyloglucoside (mg 100 g^−1^)	2.05 ± 0.09 b	4.86 ± 0.20 c	4.72 ± 0.27 c	1.09 ± 0.18 a	1.31 ± 0.17 a
	Phloridzin (mg 100 g^−1^)	2.59 ± 0.18 a	8.14 ± 0.12 d	5.55 ± 0.16 c	4.85 ± 0.14 b	4.67 ± 0.12 b
	Total dihydrochalcones (mg 100 g^−1^)	4.63 ± 0.27 a	13.00 ± 0.32 d	10.27 ± 0.11 c	5.94 ± 0.32 b	5.98 ± 0.29 b
Phenolic acids	Chlorogenic acid (mg 100 g^−1^)	4.38 ± 0.31 a	16.80 ± 0.10 c	15.44 ± 0.85 c	7.76 ± 0.34 b	6.40 ± 0.09 b
	Chlorogenic acid derivative (mg 100 g^−1^)	1.41 ± 0.14 b	0.00 ± 0.00 a	0.00 ± 0.00 a	3.30 ± 0.08 c	0.00 ± 0.00 a
	Total phenolic acids (mg 100 g^−1^)	5.79 ± 0.45 a	16.8 ± 0.10 d	15.44 ± 0.85 d	11.06 ± 0.42 c	6.40 ± 0.09 a
Flavonols	Quercetin-galactoside (mg 100 g^−1^)	1.51 ± 0.12 bc	1.21 ± 0.09 ab	0.83 ± 0.12 a	1.84 ± 0.01 cd	2.21 ± 0.15 d
	Quercetin-glucoside (mg 100 g^−1^)	1.23 ± 0.17 a	0.43 ± 0.07 b	0.14 ± 0.01 b	0.32 ± 0.05 b	0.22 ± 0.04 b
	Quercetin-xyloside (mg 100 g^−1^)	0.32 ± 0.01 a	0.29 ± 0.02 ab	0.22 ± 0.04 ab	0.27 ± 0.06 ab	0.17 ± 0.00 b
	Quercetin-arabinoside (mg 100 g^−1^)	1.37 ± 0.24 a	1.21 ± 0.08 ab	0.73 ± 0.08 b	0.26 ± 0.06 c	1.07 ± 0.08 ab
	Quercetin-rhamnoside (mg 100 g^−1^)	0.70 ± 0.08 a	2.61 ± 0.10 b	2.24 ± 0.22 b	0.89 ± 0.06 a	0.65 ± 0.09 a
	Total flavonols (mg 100 g^−1^)	5.13 ± 0.62 ab	5.75 ± 0.36 b	4.17 ± 0.08 bc	3.58 ± 0.21 c	4.32 ± 0.03 bc
Anthocyanins	Cyanidin-3-galactoside (mg 100 g^−1^)	31.74 ± 0.88 b	20.86 ± 0.52 c	32.21 ± 2.34 b	58.39 ± 0.06 a	12.12 ± 0.30 d
	Unidentified (mg 100 g^−1^)	5.79 ± 0.21 a	0.00 ± 0.00 c	6.29 ± 0.23 a	6.08 ± 0.16 a	1.40 ± 0.03 b
	Total anthocyanins (mg 100 g^−1^)	37.52 ± 1.13 b	20.86 ± 0.52 c	38.50 ± 2.11 b	64.47 ± 0.00 a	13.52 ± 0.33 d
Total phenolic compounds (mg 100 g^−1^)		56.14 ± 2.76 c	63.20 ± 1.21 c	74.57 ± 3.15 b	99.70 ± 2.16 a	32.91 ± 0.98 d

a,b,c,d—The same letters in the rows mean no statistical differences between apple samples.

**Table 3:** The mean values of selected image textures of red-fleshed apples of the cultivar ‘Trinity’ and clones 90, 120, 156, and 158.

Image Textures	Apple Sample				
	‘Trinity’	Clone 90	Clone 120	Clone 156	Clone 158
LS5SH3DifVarnc	5.00 ± 0.64 a	3.63 ± 0.33 b	3.68 ± 0.29 b	4.03 ± 0.14 b	3.65 ± 0.46 b
LS5SN5Contrast	11.57 ± 1.02 a	9.35 ± 0.37 c	9.07 ± 0.42 c	10.47 ± 0.56 b	9.05 ± 0.51 c
RS5SV1DifVarnc	3.11 ± 0.31 ab	2.30 ± 0.43 bc	1.96 ± 0.36 c	2.19 ± 0.44 bc	3.43 ± 0.35 a
LS5SN5DifVarnc	6.09 ± 0.22 a	4.69 ± 0.14 b	4.97 ± 0.19 b	5.60 ± 0.30 a	4.80 ± 0.20 b
GS5SZ5DifVarnc	5.91 ± 0.30 a	4.42 ± 0.35 b	4.63 ± 0.20 b	5.61 ± 0.27 a	4.44 ± 0.24 b
RS5SH5Correlat	0.66 ± 0.23 a	0.70 ± 0.22 ab	0.69 ± 0.21 a	0.77 ± 0.14 b	0.66 ± 0.20 a
RS5SN5SumAverg	32.83 ± 0.21 a	32.89 ± 0.20 a	32.85 ± 0.29 a	32.86 ± 0.18 a	32.85 ± 0.31 a
RS5SN3SumAverg	32.78 ± 0.24 a	32.84 ± 0.18 a	32.83 ± 0.20 a	32.79 ± 0.21 a	32.80 ± 0.33 a
RS5SH5SumAverg	32.77 ± 0.22 a	32.82 ± 0.21 a	32.80 ± 0.29 a	32.79 ± 0.17 a	32.77 ± 0.29 a
VS4RHShrtREmp	0.30 ± 0.05 a	0.24 ± 0.05 b	0.21 ± 0.07 b	0.23 ± 0.04 b	0.22 ± 0.07 b
XS5SH5Entropy	2.31 ± 0.07 a	2.30 ± 0.06 a	2.26 ± 0.08 ab	2.29 ± 0.09 ab	2.23 ± 0.07 b
SS5SH5Contrast	5.74 ± 0.26 a	5.10 ± 0.39 ab	4.68 ± 0.17 b	3.53 ± 0.12 c	4.94 ± 0.54 ab
XHPerc10	60.07 ± 1.65 a	67.56 ± 2.03 b	67.54 ± 1.98 b	60.57 ± 2.54 a	59.94 ± 3.50 a
SS5SH3SumEntrp	1.48 ± 0.13 a	1.49 ± 0.09 a	1.46 ± 0.12 a	1.45 ± 0.13 a	1.46 ± 0.16 a

a,b,c—The same letters in the rows mean no statistical differences between apple samples.

**Table 4:** The correlations between image texture parameters with sugars and acids of red-fleshed apples belonging to the cultivar ‘Trinity’ and clones 90, 120, 156, and 158.

Properties	Strongest Positive Correlations		Strongest Negative Correlations	
	Textures	Correlation Coefficients (R)	Textures	Correlation Coefficients (R)
Sucrose (g kg^−1^)	LS5SH3DifVarnc	0.996	RSGSkewness	−0.981
Glucose (g kg^−1^)	GS5SH3SumAverg	0.994	LS5SZ1SumOfSqs	−0.974
Fructose (g kg^−1^)	GS5SN1SumAverg	0.987	aS4RNGLevNonU	−0.992
Sorbitol (g kg^−1^)	LS5SN5Contrast	0.993	GSGKurtosis	−0.997
Total sugars (g kg^−1^)	RS5SV1DifVarnc	0.975	GS5SH1SumVarnc	−0.998
L-ascorbic acid (mg 100 g^−1^)	LS5SN5DifVarnc	0.990	ZS5SV3SumEntrp	−0.976
Malic acid (mg 100 g^−1^)	GS5SZ5DifVarnc	0.991	YHPerc10	−0.984
Citric acid (mg 100 g^−1^)	LS5SV3Contrast	0.995	ZS5SH3Correlat	−0.999

**Table 5:** The correlations between image textures with phenolic compounds of red-fleshed apples of the cultivar ‘Trinity’ and clones 90, 120, 156, and 158.

Phenolic Compounds (mg 100 g^−1^)	Strongest Positive Correlations		Strongest Negative Correlations	
	Textures	Correlation Coefficients (R)	Textures	Correlation Coefficients (R)
Procyjanidin B1	RS5SH3SumAverg	0.996	bS5SZ1Contrast	−0.970
Procyjanidin B2	RS5SH5Correlat	0.998	US5SZ5SumEntrp	−0.995
Epicatechin	ZS4RVLngREmph	0.987	US5SN5SumEntrp	−0.978
Total flavanols	RS5SH5Correlat	0.999	US5SN5SumEntrp	−0.996
Phloretin xyloglucoside	bS5SN1Correlat	0.989	ZHKurtosis	−0.958
Phloridzin	RS5SN5SumAverg	0.967	XS5SZ5DifVarnc	−0.906
Total dihydrochalcones	RS5SN3SumAverg	0.985	aS5SV1SumAverg	−0.952
Chlorogenic acid	RS5SN3SumAverg	0.981	aS5SV1SumAverg	−0.948
Chlorogenic acid derivative	bHKurtosis	0.998	aS5SH5SumOfSqs	−0.998
Total phenolic acids	RS5SH5SumAverg	0.950	LHKurtosis	−0.949
Quercetin-galactoside	bS5SV1Contrast	0.971	bS5SV1Correlat	−0.996
Quercetin-glucoside	VS4RHShrtREmp	0.996	LS5SH3Correlat	−0.980
Quercetin-xyloside	XS5SH5Entropy	0.999	SS5SV5AngScMom	−0.991
Quercetin-arabinoside	SS5SH5Contrast	0.973	US5SV3AngScMom	−0.963
Quercetin-rhamnoside	XHPerc10	0.990	bS5SZ1Contrast	−0.947
Total flavonols	SS5SH3SumEntrp	0.997	VHMaxm01	−0.911
Cyanidin-3-galactoside	bATeta1	0.988	UHPerc99	−0.988
Unidentified anthocyanins	RHPerc01	0.952	BS5SV3Entropy	−0.986
Total anthocyanins	bATeta1	0.972	UHPerc99	−0.972

**Table 6:** Regression equations for sugars and acids of red-fleshed apples of the cultivar ‘Trinity’ and clones 90, 120, 156, and 158.

Regression Equations	Coefficients of Determination (R^2^)	F	df	*p*
Sucrose (g kg^−1^) = −26.41 + 14.937 × LS5SH3DifVarnc	0.992	372.4444	1.3	0.000304
Glucose (g kg^−1^) = −3422.00 + 106.25 × GS5SH3SumAverg	0.989	261.2623	1.3	0.000515
Fructose (g kg^−1^) = 69.727 − 0.0032 × aS4RNGLevNonU	0.985	193.9769	1.3	0.000801
Sorbitol (g kg^−1^) = 11.244 − 0.3047 × GSGKurtosis	0.994	518.8034	1.3	0.000185
Total sugars (g kg^−1^) = 537.37 − 4.493 × GS5SH1SumVarnc	0.996	781.1450	1.3	0.000101
L-ascorbic acid (mg 100 g^−1^) = −21.31 + 4.9591 × LS5SN5DifVarnc	0.981	156.4119	1.3	0.001102
Malic acid (mg 100 g^−1^) = −10.84 + 4.5163 × GS5SZ5DifVarnc	0.982	161.2866	1.3	0.001053
Citric acid (mg 100 g^−1^) = 86.706 − 86.35 × ZS5SH3Correlat	0.998	1578.295	1.3	0.000035

**Table 7:** Regression equations for phenolic compounds (mg 100 g^−1^) of red-fleshed apples of the cultivar ‘Trinity’ and clones 90, 120, 156, and 158.

Regression Equations	Coefficients of Determination (R^2^)
Procyjanidin B1 = −683.50 + 20.887 × RS5SH3SumAverg	0.992
Procyjanidin B2 = −23.49 + 38.597 × RS5SH5Correlat	0.996
Epicatechin = 0.99440 + 0.01605 × ZS4RVLngREmph	0.974
Total flavanols = −68.34 + 108.07 × RS5SH5Correlat	0.998
Phloretin xyloglucoside = −254.10 + 278.71 × bS5SN1Correlat	0.979
Phloridzin = −2935.00 + 89.470 × RS5SN5SumAverg	0.935
Total dihydrochalcones = −4647.00 + 141.89 × RS5SN3SumAverg	0.971
Chlorogenic acid = −7340.00 + 224.06 × RS5SN3SumAverg	0.963
Chlorogenic acid derivative = 28.131 − 1.075 × aS5SH5SumOfSqs	0.995
Total phenolic acids = −7702.00 + 235.23 × RS5SH5SumAverg	0.903
Quercetin-galactoside = 55.779 − 57.39 × bS5SV1Correlat	0.992
Quercetin-glucoside = −2.536 + 12.385 × VS4RHShrtREmp	0.993
Quercetin-xyloside = −3.793 + 1.7772 × XS5SH5Entropy	0.998
Quercetin-arabinoside = −1.624 + 0.53193 × SS5SH5Contrast	0.947
Quercetin-rhamnoside = −13.03 + 0.22878 × XHPerc10	0.980
Total flavonols = −68.93 + 50.147 × SS5SH3SumEntrp	0.994
Cyanidin-3-galactoside = 796.18 − 6.528 × UHPerc99	0.976
Unidentified anthocyanins = 6.0582 − 0.2 × 10^−3^ × BS5SV3Entropy	0.972
Total anthocyanins = −568.90 + 1034.40 × bATeta1	0.945

## Data Availability

The original contributions presented in this study are included in the article. Further inquiries can be directed to the corresponding author.
